# Prognostic Significance of β-Catenin, E-Cadherin, and SOX9 in Colorectal Cancer: Results from a Large Population-Representative Series

**DOI:** 10.3389/fonc.2014.00118

**Published:** 2014-05-21

**Authors:** Jarle Bruun, Matthias Kolberg, Jahn M. Nesland, Aud Svindland, Arild Nesbakken, Ragnhild A. Lothe

**Affiliations:** ^1^Department for Cancer Prevention, Institute for Cancer Research, Norwegian Radium Hospital, Oslo University Hospital, Oslo, Norway; ^2^Centre for Cancer Biomedicine, Faculty of Medicine, University of Oslo, Oslo, Norway; ^3^Department of Pathology, Oslo University Hospital, Oslo, Norway; ^4^Faculty of Medicine, University of Oslo, Oslo, Norway; ^5^Department of Gastrointestinal Surgery, Aker Hospital, Oslo University Hospital, Oslo, Norway; ^6^Department of Molecular Biosciences, Faculty of Mathematics and Natural Sciences, University of Oslo, Oslo, Norway

**Keywords:** beta-catenin, E-cadherin, SOX9 transcription factor, prognostic biomarkers, colorectal cancer, biomarker discovery, guideline adherence

## Abstract

Robust biomarkers that can precisely stratify patients according to treatment needs are in great demand. The literature is inconclusive for most reported prognostic markers for colorectal cancer (CRC). Hence, adequately reported studies in large representative series are necessary to determine their clinical potential. We investigated the prognostic value of three Wnt signaling-associated proteins, β-catenin, E-cadherin, and SOX9, in a population-representative single-hospital series of 1290 Norwegian CRC patients by performing immunohistochemical analyses of each marker using the tissue microarray technology. Loss of membranous or cytosolic β-catenin and loss of cytosolic E-cadherin protein expression were significantly associated with reduced 5-year survival in 903 patients who underwent major resection (722 evaluable tissue cores) independently of standard clinicopathological high-risk parameters. Pre-specified subgroup analyses demonstrated particular effect for stage IV patients for β-catenin membrane staining (*P* = 0.018; formal interaction test *P* = 0.025). Among those who underwent complete resection (714 patients, 568 evaluable), 5-year time-to-recurrence analyses were performed, and stage II patients with loss of cytosolic E-cadherin were identified as an independent high-risk subgroup (*P* = 0.020, formal interaction test was not significant). Nuclear β-catenin and SOX9 protein, regardless of intracellular location, were not associated with prognosis. In conclusion, the protein expression level of membranous or cytosolic β-catenin and E-cadherin predicts CRC patient subgroups with inferior prognosis.

## Introduction

As of 2008, 1.2 million patients were diagnosed with colorectal cancer (CRC) annually, and only about half of them were alive 5 years after their initial diagnosis (1). Clinicopathological staging is the best available system to predict disease course, however, the system offers only crude estimates leading to unnecessary treatment of a large number of patients on one hand, and recurrence of disease among patients who only received surgery, on the other hand. Taken together with the fact that CRC risk increases with age and that the world’s population is both growing and aging, the coming decades will put an unprecedented pressure on health care institutions worldwide (2). Therefore, the need for molecular biomarkers to guide clinical decision-makers on how to stratify patients into optimal treatment regiments has never been greater. In particular, patients with stage II CRC are not routinely offered adjuvant therapy, although about 20–30% of them experience relapse and die within 5 years after surgery (3). Also, stage III patients above 75 years of age are not routinely offered adjuvant therapy although evidence suggests a benefit from such treatment (4, 5). Prognostic biomarkers that distinguish between both high-risk and low-risk patients within these stages are highly warranted.

In the early 90s, the hereditary cancer syndrome familial adenomatous polyposis (FAP) was discovered to be directly linked to mutations within the adenomatous polyposis coli gene (APC) (6, 7). Two years later, a close interaction between APC and β-catenin was demonstrated (8, 9), and as APC mutations were found at high frequencies in colorectal adenomas and carcinomas, it was soon realized that the Wnt/β-catenin signaling pathway plays an initiating and rate-limiting role in colorectal tumorigenesis (10–12). More recently, large-scale exome-sequencing efforts have confirmed that Wnt/β-catenin signaling is deregulated in more than 90% of all CRCs (13–15).

Briefly, canonical Wnt signaling (i.e., Wnt/β-catenin signaling) is initiated when Wnt proteins are released by stromal cells and Paneth cells in the intestinal crypt, and these proteins bind to heterodimeric receptor complexes on the surface of intestinal stem cells (Frizzled/Lrp6) and their immediate descendants (16). A signal is then conveyed along a signaling cascade, which essentially inhibits degradation of cytoplasmic β-catenin. β-Catenin then soon translocates into the nucleus where it interacts with DNA-bound TCF/Lef transcription factors, causing expression of a range of genes related to proliferation and differentiation, including SOX9. In cancer cells, mutations in APC, β-catenin, or AXIN 2 cause constitutive activation of this signaling pathway, leading to excess proliferation and inhibition of differentiation of stem cell progenitors. Notably, β-catenin also serves another essential cellular function in adherens junctions by linking E-cadherin to the cytoskeleton, and recent evidence suggests that this β-catenin pool is highly stable and unrelated to its impact on Wnt signaling (16).

The prognostic potential of various components of the Wnt/β-catenin pathway in CRC has been explored in many datasets over the last decade, both on the genetic and the protein level. In particular, deregulation of APC, β-catenin, and E-cadherin has received much attention (17–19). From the very high frequency of APC mutations in sporadic CRC, at around 70–80% (20), it follows that its prognostic potential is likely limited. Indeed, few reports have documented robust clinical relevance of this biomarker, neither at the genetic level (21) nor at the protein level (22), although reports suggest that mutations affecting β-catenin-binding sites may have prognostic value (13, 23). In contrast, the literature on β-catenin and E-cadherin has been clouded by many conflicting findings due to a large number of unstandardized and underpowered studies (17, 18, 21, 24–38), and their potential as biomarkers in CRC still merits further investigation.

SOX9 is a transcription factor and a downstream target of Wnt/β-catenin signaling with possible roles in β-catenin regulation (39–42). Deregulation of SOX9 has been reported for several cancers, including CRC (24), and recent large-scale exome-sequencing efforts have revealed that SOX9 is mutated in a subset of CRCs (15). The prognostic potential of SOX9 has only been evaluated in one adequate CRC dataset, which suggested that high expression of the SOX9 protein was associated with an adverse prognosis (43).

Here, we used a tissue microarray (TMA) constructed from a large consecutive, population-representative single-hospital series of primary CRCs to explore the prognostic significance of the protein expression of β-catenin, E-cadherin, and SOX9 by immunohistochemistry. We have attempted to report the study according to the REMARK guidelines (Table S1 in Supplementary Material) (44) and primarily focused on the clinical relevance within CRC stages. We specifically sought to test the hypotheses that (i) increased nuclear β-catenin staining is associated with poor outcome, indicating active Wnt/β-catenin signaling, that (ii) loss of β-catenin and E-cadherin membrane staining is associated with poor outcome due to decoupling of adherence junctions in epithelial–mesenchymal transition (EMT), and whether (iii) differential expression of the downstream Wnt signaling target, SOX9, identifies prognostic subgroups of CRC patients.

## Materials and Methods

### Patient material

**Figure 1:**
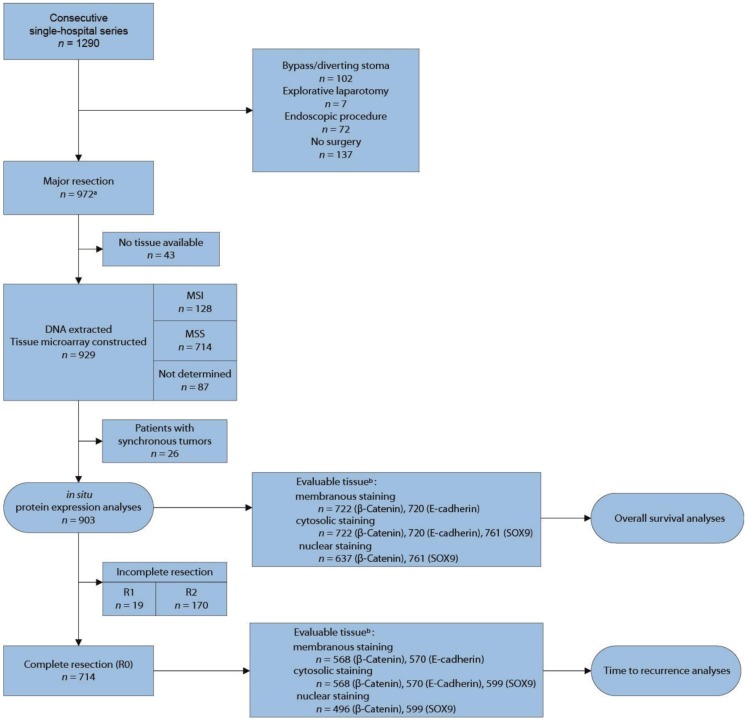
**Flow diagram for inclusion of patients in the study**. ^a^Tissue from four patients with endoscopic procedure included. ^b^Unevaluable tissue had insufficient number of epithelial tumor cells, extensive necrosis, and/or poor tumor preservation.

A population-representative consecutive series of 1290 CRC patients admitted to Oslo University Hospital – Aker (1993–2003) was analyzed. This hospital treated all CRC patients from a geographically defined catchment area, including most relapses. Of these, 929 patients underwent major resection, and DNA was extracted from corresponding formalin-fixed paraffin-embedded (FFPE) tissue from which a TMA was constructed (Figure 1). Major resection was defined as removal of the tumor-bearing bowel segment with the lymphovascular pedicle and mesentery. All rectal cancers were surgically removed by total mesorectal excision (TME). Resection status was defined as R0 (complete resection/no residual tumor), R1 (microscopic residual cancer at the resection margin), or R2 (macroscopic or radiological evidence of residual cancer, locally or distant). TNM-staging and histopathological grading followed the UICC/AJCC system, version 5. Comprehensive clinical data had been prospectively registered on all patients (Table 1). Microsatellite instability (MSI) status was previously determined for all tumors (3) (Bruun et al., unpublished). More than 95% of patients were of Caucasian ethnicity (based on name-origin). Additional clinical information has been reported elsewhere (3, 45).

**Table 1:** **Patient characteristics for all patients included in the study**.

Patient characteristic	Frequency (*n*)	Percentage (%)
Patients in the study	903	100
**AGE**		
Median	73	
Range	30–94	
**AGE (3 GROUPS, BINNED)**		
30–68	309	34.2
69–77	292	32.3
78–94	302	33.4
**GENDER**		
Male	429	47.5
Female	474	52.5
**STAGE**		
I	133	14.8
II	363	40.4
III	237	26.4
IV	165	18.4
ND	4	–
**HISTOPATHOLOGIC GRADE**		
G1	84	9.6
G2	674	76.8
G3	108	12.3
Mucinous^a^	12	1.4
ND^a^	25	–
**TUMOR LOCATION**		
Proximal colon	367	40.6
Distal colon	302	33.4
Rectum	234	25.9
**RESECTION**		
0	713	79.0
1	19	2.0
2	170	18.8
ND^a^	1	–
**MICROSATELLITE INSTABILITY**		
MSI	119	14.5
MSS	700	85.5
ND^a^	84	–

*^a^Excluded from statistical analyses. ND, no data*.

The study was approved by the Regional Committee for Medical and Health Research Ethics, South-Eastern Norway (REK number 1.2005.1629) and the Norwegian Data Protection Authority, and the patients were enrolled after informed consent. The research conformed to the Declaration of Helsinki and the research biobanks have been registered according to national legislation.

### Immunohistochemical analyses of protein expression on TMA

Cores from FFPE tissue from 670 colonic, 233 rectal, and 26 synchronous carcinomas (one 0.6 mm diameter core per patient taken from a viable, non-necrotic tumor area) from patients treated at Oslo University Hospital – Aker (1993–2003), were organized into a TMA according to the original method described by Kononen and colleagues in 1998 (46).

The immunohistochemical analyses were done on 3–4 μm thick TMA sections on microscope slides, and were performed as previously described (47). Briefly, sections were de-paraffinized in xylene for 10 min, and then rehydrated. Antigen retrieval was performed in a microwave oven by heating the sections in plastic containers filled with Tris/EDTA-buffer (pH 9). Staining was performed according to the DAKO Envision protocol, using the reagents supplied with the K5007 kit (Dako, Glostrup, Denmark). Immunocomplexes were visualized with the chromogenic stain diaminobenzidine (DAB). Hematoxylin staining was used to visualize the nuclear compartment. A test TMA containing representative tissues from nine human organs and six types of cancer was utilized to optimize staining conditions to the dynamic range of DAB by careful titration of antibodies. To evaluate non-specific secondary antibody reactions, a negative control experiment was provided by omitting the primary antibody from one slide.

For immunohistochemical analysis, mouse monoclonal anti-β-catenin (Clone 14) antibodies were obtained from BD Biosciences (San Jose, CA, USA; Catalog number 610154), recognizing a C-terminal epitope between residue 571 and residue 781 of β-catenin. Mouse monoclonal anti-E-cadherin (Clone 36) antibodies were obtained from BD Biosciences (catalog number 610181), recognizing a C-terminal epitope between residue 735 and residue 883 of E-cadherin. Rabbit polyclonal anti-SOX9 antibodies were obtained from Atlas antibodies AB (catalog number HPA001758, Stockholm, Sweden), recognizing the 117 amino acid residue C-terminal end. The antibodies were employed at dilutions of 1:800, 1:2000, and 1:500, respectively.

### Evaluation of immunostaining

The staining of β-catenin, E-cadherin, and SOX9 was scored according to the proportion and intensity categories proposed by Allred et al. (48). The proportion score represents the estimated fraction of positive cells (0 = none, 1 = <1%, 2 = 1–10%, 3 = 11–33%, 4 = 34–66%, and 5 = 67–100%), while the intensity score represents their average staining intensity (0 = negative, 1 = weak, 2 = intermediate, and 3 = strong). The final Allred-score for each tumor is calculated by adding these two scores. Staining was evaluated and scored separately for membranous, cytoplasmic, and nuclear staining patterns. The scores were combined into strong, moderate, and weak categories. The categories were determined by the number of patients and events in each subgroup, and the ability to visually differentiate reliably between the staining scores. All analyses were done in parallel on ungrouped scores demanding that findings were valid for both ungrouped and grouped data. The scoring was performed independently by two investigators (Jarle Bruun and Matthias Kolberg), blinded to clinical data, in close collaboration with an experienced pathologist (Jahn M. Nesland). All discrepancies were resolved and reassigned on consensus of opinion.

For β-catenin the interobserver agreement, as measured by the intraclass correlation coefficients (ICC) (49) were 0.88, 0.89, and 0.85 for membranous, cytosolic, and nuclear staining, respectively; for E-cadherin 0.84 and 0.67 for membranous and cytosolic staining, respectively; for SOX9 0.93 for nuclear staining. Due to the limited ability of the Allred scoring system to differentiate proportionately between negative tumors (score 0) and weak tumors (score 4–6, mostly), these ICCs underestimate the true ICC value, especially for cytosolic staining for which tumors largely exhibited a uniform staining with proportion scores of 4 or 5. Calculations were confirmed by cross-tabular visualizations.

### Statistical analyses

Five-year overall survival (OS) and time-to-recurrence (TTR) plots were generated using the Kaplan–Meier method in the SPSS 18.0 software (SPSS, IL, USA). TTR and OS were defined according to the guidelines given by Punt et al. (50). Briefly, TTR was defined as the time from surgery to the first event of either death from the same cancer, local recurrence, or distant metastasis. Patients were censored at death from other cancer, non-cancer death, post-operative death (<3 months), and loss to follow-up. OS was defined as the time from surgery to death from any cause, and patients were censored at loss to follow-up. No patients were lost to follow-up in the study period. The logrank test for trend was used to compare survival between ordinal groups, and Cox proportional hazards regression modeling (Wald test) was used to provide univariate and multivariate hazard-ratios (HR) and confidence intervals (CI). Age categories were created by three-tire binning to achieve sufficient statistical strength within each category. In multivariate analyses, the protein parameters with significant independent impact on patient survival were adjusted for the standard and high-risk clinicopathological variables: age, gender, tumor stage, tumor differentiation, tumor location, MSI status, and residual tumor status. Adjuvant treatment for patients with stage III colon cancer (<75 years of age) became standard treatment in 1997 and was considered in initial multivariate models. These patients were few and adjustment did not affect the models. Adjustment for pre- and post-operative radiotherapy for rectal cancer patients was also considered, but was pertinent to only a very limited number of patients and therefore not included in initial models. The proportional hazards assumptions were verified by graphical evaluation of plots of log (−log survival time) versus log time.

The correlation and survival analyses involve multiple tests and false positive findings are to be expected with a 5% significance level. However, several of the clinicopathological parameters, such as stage and differentiation, can be assumed to have some *a priori* association with the three biomarkers, reducing the need for rigorous correction. Clinically relevant subgroup analyses were therefore pre-specified and additional subgroup analyses labeled as exploratory. Interaction tests were integrated in the Cox models to assess whether effects were different between subgroups, but must be interpreted carefully due to the low power of such tests. All *P*-values were two-sided and derived from statistical tests using SPSS, and considered statistically significant at *P* < 0.05. To correct for multiple testing in the correlation analyses, we set a significance threshold of *P* < 0.001. Correlation between expression of protein markers and standard clinicopathological variables was evaluated using Spearman’s rho test.

## Results

### TMA immunostaining results

**Figure 2:**
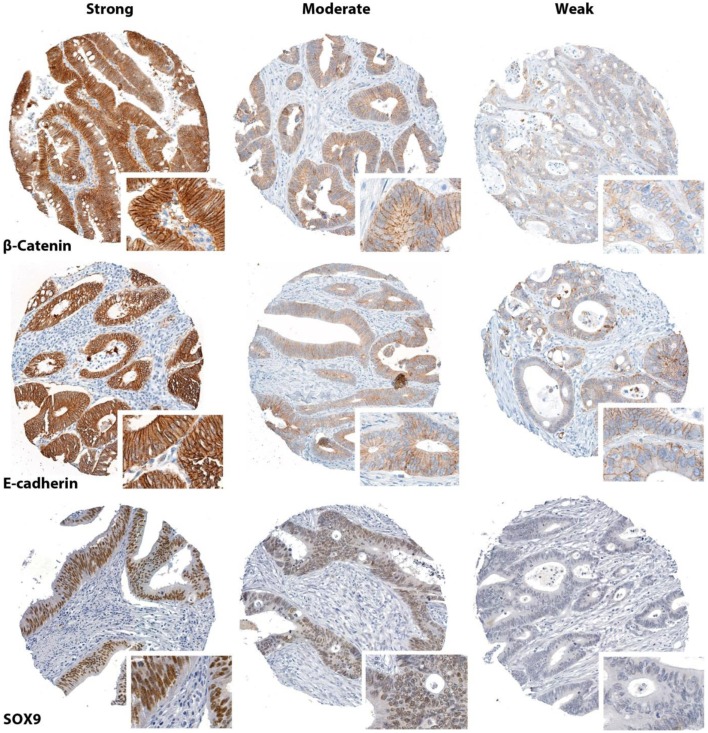
**Representative immunostaining patterns for P-catenin, E-cadherin, and SOX9 (core diameter 0.6 mm)**.

Among 903 stained histospots, 722 (80%) were evaluable for β-catenin, 720 (80%) for E-cadherin, and 761 (84%) for SOX9 (Figure 2). The rest of the histospots were unevaluable due to insufficient number of epithelial tumor cells, extensive necrosis, poor tumor preservation, or loss of tissue on the TMA slide. Eighty-five samples with very strong cytoplasmic β-catenin staining could not be evaluated for nuclear staining, leaving 637 (71%) for this purpose. No bias of clinical data was observed for these 85 samples. Generally, the tumor exhibited various degrees of staining for all the three proteins in the compartments investigated (Figure S1 in Supplementary Material).

### Correlation between immunostaining and with clinicopathological variables

Membrane and cytosolic β-catenin staining correlated strongly with E-cadherin staining, in accordance with their common functional roles in adherens junctions (Table 2). Nuclear β-catenin, however, correlated only with cytosolic β-catenin, but not to β-catenin membrane staining, E-cadherin, or SOX9.

**Table 2:** **Correlation between staining of studied biomarkers**.

Marker	β-Catenin (*P, r, n*)			E-cadherin (*P, r, n*)		SOX9 (*P, r, n*)	
	Cytosol	Membrane	Nucleus	Cytosol	Membrane	Cytosol	Nucleus
**β-CATENIN**							
Cytosol	1	6.6 × 10^−25^	1.2 × 10^−49^	9.7 × 10^−26^	5.3 × 10^−22^	2.0 × 10^−10^	1.3 × 10^−5^
		0.37	0.54	0.38	0.35	0.24	0.16
		722	637	702	702	700	700
Membrane		1	0.0079	3.0 × 10^−32^	2.5 × 10^−63^	0.36	0.22
			−0.11	0.43	0.58	0.034	0.046
			637	702	702	702	702
Nucleus			1	0.17	0.011	0.17	0.037
				0.055	0.1	0.055	0.084
				618	618	616	616
**E-CADHERIN**							
Cytosol				1	3.1 × 10^−86^	0.57	0.42
					0.65	0.022	−0.03
					720	694	694
Membrane					1	0.48	0.064
						0.027	0.07
						694	694
**SOX9**							
Cytosol						1	3.1 × 10^−70^
							0.58
							761
Nucleus							1

**P*-values and correlation coefficients (*r*) from Spearman’s rho test. The correlations are calculated using ungrouped Allred staining scores*.

All the tested biomarkers in all subcellular locations were more highly expressed in MSS tumors than in MSI, except for β-catenin membrane and nuclear SOX9 staining, but these showed the same trend (Table 3). Similar, but weaker associations were found for left-sided tumors (including rectum) as compared to right-sided tumors (Table 3). Histopathologic grade was also positively correlated with expression of all biomarkers. The correlation was weaker for β-catenin membrane and nuclear staining, and SOX9 nuclear staining, but these showed the same trend.

**Table 3:** **Correlation between studied biomarkers and patient characteristics**.

Patient characteristic	β-Catenin (*P, r, n*)			E-cadherin (*P, r, n*)		SOX9 (*P, r, n*)	
	Cytosol	Membrane	Nucleus	Cytosol	Membrane	Cytosol	Nucleus
Age	0.60–0.020	0.83	0.81	0.45	0.57	0.097	0.033
	722	0.0078	0.01	−0.028	0.021	0.06	0.077
		722	637	720	720	761	761
**GENDER**							
Female = 1 Male = 2	0.036	0.4	0.025	0.072	0.028	0.85	0.43
	0.078	0.031	0.089	0.067	0.082	−0.0069	−0.029
	722	722	637	720	720	761	761
Stage	0.081	0.043	0.3	0.013	0.088	0.035	0.74
	−0.065	−0.076	0.041	−0.093	−0.064	−0.077	−0.012
	718	718	633	716	716	757	757
**HISTOPATHOLOGIC GRADE**							
G1, G2, G3	2.4 × 10^−6^	0.014	0.14	8.0 × 10^−6^	2.9 × 10^−5^	7.1 × 10^−5^	0.057
	0.18	0.093	0.059	0.17	0.16	0.15	0.07
	694	694	612	691	691	731	731
**TUMOR LOCATION**							
Right = 1	2.8 × 10^−9^	0.17	1.2 × 10^−6^	3.8 × 10^−7^	2.2 × 10^−6^	0.13	0.11
Left = 2	0.22	0.051	0.19	0.19	0.18	0.055	−0.059
Rectum = 3	722	722	637	720	720	761	761
**RESECTION**							
R0, R1, R2	0.028	0.2	0.48	0.045	0.35	0.027	0.93
	−0.082	−0.047	0.028	−0.075	−0.035	−0.08	−0.0032
	721	721	636	719	719	760	760
MSI status	3.6 × 10^−21^	0.012	1.1 × 10^−12^	2.6 × 10^−9^	1.5 × 10^−9^	1.8 × 10^−6^	0.091
MSI = 1	0.36	0.097	0.29	0.23	0.23	0.18	0.064
MSS = 2	661	661	584	657	657	693	693

**P*-values and correlation coefficients (*r*) from Spearman’s rho test. Correlations are calculated using ungrouped Allred staining scores*.

There were no significant correlations with any of the clinical parameters age, gender, or tumor stage.

### Survival analysis

Univariate and multivariate OS analysis of all patients and TTR analysis of patients with complete resection were carried out in order to assess the prognostic potential of each of the three biomarkers.

### Loss of β-catenin independently predicts poor outcome

**Figure 3:**
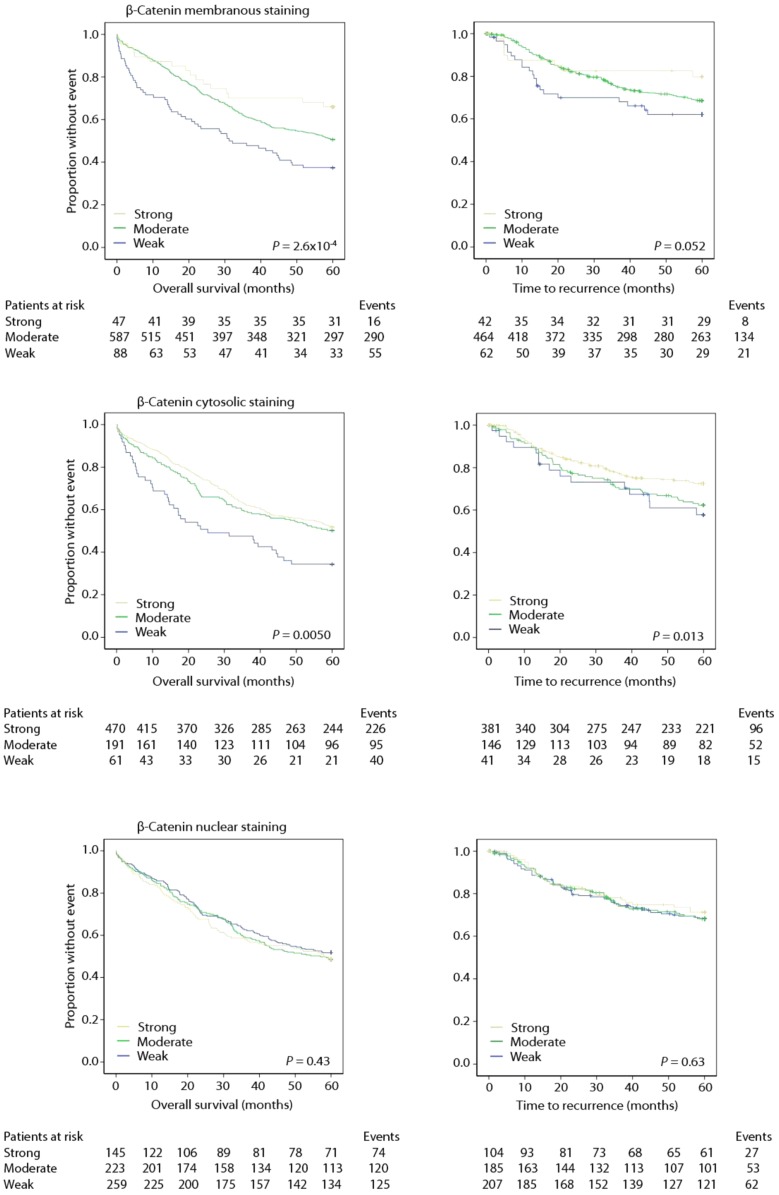
**Weak staining of β-catenin predicts poor outcome**.

Univariate analyses showed that decreased membranous staining of β-catenin was significantly associated with a worse prognosis (Figure 3; Tables 4 and 5).

**Table 4:** **Univariate and multivariate modeling by Cox regression (Wald test), overall survival (OS)**.

Variable	Univariate						Multivariate				
	*P*^a^	HR	95%	*P*	*n*	Events	HR	95% CI	*P*	*n*	Events
Age^b^	–	1.027	1.019–1.036	5.35 × 10^−10^	903	449	1.036	1.027–1.046	2.0 × 10^−13^		
**GENDER**											
Female		1					1				
Male	0.4	0.92	0.77–1.11	0.4	903	449	1.1	0.88–1.33	0.48		
**STAGE**											
I		1					1				
II		1.89	1.27–2.80				1.61	1.05–2.46			
III		3.06	2.06–4.57				2.83	1.85–4.33			
IV	1.6 × 10^−56^	11.5	7.77–17.2	1.7 × 10^−63^	898	446	3.91	2.22–6.89	1.6 × 10^−8^		
**HISTOPATHOLOGIC GRADE**											
G1		1					1				
G2		1.49	1.03–2.16				1.21	0.81–1.80			
G3	1.6 × 10^−7^	2.8	1.84–4.27	2.0 × 10^−7^	866	433	2.33	1.46–3.71	1.3 × 10^−5^		
**TUMOR LOCATION**											
Proximal colon		1					1				
Distal colon		1.01	0.82–1.25				0.95	0.75–1.21			
Rectum	0.0061	0.69	0.54–0.88	0.0048	903	449	0.9	0.068–1.20	0.76		
**RESECTION**											
0		1					1				
1		2.03	1.11–3.71				1.7	0.84–3.45			
2	6.2 × 10^−85^	6.17	5.03–7.56	2.88 × 10^−67^	902	448	3.47	2.31–5.21	8.4 × 10^−9^		
**MICROSATELITE STATUS**											
MSI		1					1				
MSS	0.039	1.37	1.01–1.84	0.04	819	412	1.69	1.18–2.42	0.0043	787	396
**β-CATENIN MEMBRANE STAINING^c^**											
Weak		1					1				
Moderate		0.64	0.48–0.85				0.61	0.45–0.83			
Strong	2.6 × 10^−4^	0.41	0.23–0.71	0.0012	722	361	0.54	0.29–0.99	0.0065	637	321
**β-CATENIN CYTOSOLIC STAINING^c^**											
Weak		1					1				
Moderate		0.61	0.42–0.89				0.6	0.40–0.89			
Strong	0.005	0.56	0.40–0.79	0.0038	722	361	0.64	0.44–0.93	0.033	637	321
**β-CATENIN NUCLEAR STAINING^c^**											
Weak		1					1				
Moderate		1.09	0.85–1.40				0.99	0.75–1.30			
Strong	0.43	1.11	0.83–1.48	0.7	637	319	0.97	0.71–1.34	0.99	562	282
**E-CADHERIN MEMBRANE STAINING^c^**											
Weak		1					1				
Moderate		0.97	0.70–1.36				0.93	0.63–1.36			
Strong	0.1	0.82	0.60–1.12	0.23	720	355	0.79	0.54–1.15	0.3	633	315
**E-CADHERIN CYTOSOLIC STAINING^c^**											
Weak		1					1				
Moderate		0.8	0.64–0.99				0.93	0.73–1.18			
Strong	0.0014	0.69	0.48–1.00	0.045	720	355	0.85	0.57–1.28	0.69	633	315
**SOX9 CYTOSOLIC STAINING^c^**											
Weak		1					1				
Moderate		0.87	0.70–1.08				1.06	0.83–1.34			
Strong	0.5	0.99	0.70–1.39	0.4	761	383	1.04	0.72–1.51	0.89	668	339
**SOX9 NUCLEAR STAINING^c^**											
Weak		1					1				
Moderate		0.98	0.76–1.27				1.13	0.85–1.50			
Strong	0.82	1.03	0.77–1.36	0.93	761	383	0.99	0.72–1.36	0.53	668	339

*^a^Logrank test*.

*^b^Age is implemented as a continuous variable*.

*^c^Adjusted for age, gender, stage, grade, location, resection, and microsatellite status*.

**Table 5:** **Univariate and multivariate modeling by Cox regression (Wald test), time to recurrence (TTR)**.

Variable	Univariate						Multivariate^a^				
	*P*^a^	HR	95%	*P*	*n*	Events	HR	95% CI	*P*	*n*	Events
Age^b^	–	1.017	1.005–1.029	0.0067	693	206	1.023	1.009–1.037	9.4 × 10^−4^		
**GENDER**											
Female		1					1				
Male	0.96	1.007	0.77–1.32	0.96	693	206	1.09	0.80–1.47	0.6		
**STAGE**											
I		1					1				
II		2.68	1.55–4.63				2.38	1.35–4.18			
III		4.97	2.88–8.58				4.75	2.72–8.30			
IV	2.3 × 10^−45^	9.56	4.29–21.3	5.1 × 10^−11^	689	205	8.02	3.5–18.2	3.1 × 10^−10^		
**HISTOPATHOLOGIC GRADE**											
G1		1					1				
G2		1.34	0.81–2.21				1.14	0.68–1.93			
G3		2.47	1.37–4.47	0.0024	666	200	2.29	1.21–4.31	0.0042		
**TUMOR LOCATION**											
Proximal colon		1					1				
Distal colon		1.02	0.74–1.41				0.99	0.68–1.43			
Rectum	0.82	1.04	0.74–1.45	0.98	693	206	1.27	0.86–1.88	0.37		
**MICROSATELITE STATUS**											
MSI		1					1				
MSS	0.2	1.31	0.86–1.97	0.21	627	194	1.63	0.99–2.71	0.057	603	187
**β-CATENIN MEMBRANE STAINING^c^**											
Weak		1					1				
Moderate		0.72	0.45–1.14				0.84	0.50–1.41			
Strong	0.052	0.47	0.21–1.05	0.16	553	163	0.68	0.29–1.60	0.67	489	149
**β-CATENIN CYTOSOLIC STAINING^c^**											
Weak		1					1				
Moderate		0.88	0.49–1.56				0.72	0.39–1.35			
Strong	0.013	0.61	0.35–1.05	0.043	553	163	0.55	0.30–1.01	0.1	489	149
**β-CATENIN NUCLEAR STAINING^c^**											
Weak		1					1				
Moderate		0.99	0.69–1.43				0.91	0.61–1.36			
Strong	0.63	0.89	0.56–1.39	0.86	485	142	0.86	0.53–1.41	0.82	429	129
**E-CATENIN MEMBRANE STAINING^c^**											
Weak		1					1				
Moderate		1.11	0.68–1.81				0.93	0.53–1.65			
Strong	0.1	0.79	0.49–1.26	0.12	555	164	0.79	0.45–1.39	0.55	489	150
**E-CATENIN CYTOSOLIC STAINING^c^**											
Weak		1					1				
Moderate		0.65	0.47–0.89				0.67	0.47–0.95			
Strong	9.3 × 10^−4^	0.46	0.26–0.83	0.004	555	164	0.48	0.25–0.92	0.02	489	150
**SOX9 CYTOSOLIC STAINING^c^**											
Weak		1					1				
Moderate		0.9	0.65–1.24				1.04	0.72–1.49			
Strong	0.87	1.05	0.63–1.75	0.74	583	168	1.09	0.63–1.90	0.95	512	153
**SOX9 NUCLEAR STAINING^c^**											
Weak		1					1				
Moderate		1.06	0.72–1.55				1.03	0.68–1.56			
Strong	0.52	0.88	0.56–1.37	0.61	583	168	0.79	0.48–1.29	0.41	512	153

*^a^Logrank test*.

*^b^Age is implemented as a continuous variable*.

*^c^Adjusted for age, gender, stage, grade, location, and microsatellite status*.

A multivariate Cox regression model including standard clinicopathological variables demonstrated that β-catenin membranous staining was an independent prognostic marker using OS as an endpoint (Table 4), but not using TTR as an endpoint (Table 5).

Stratification according to stage demonstrated valid significance for OS only within stage IV (*P* = 3.7 × 10^−7^, *n* = 134 with 125 events, Table 6), supported by formal interaction tests (integrated in the Cox-model) assessing the probability of subgroup effects (*P* = 0.018 for β-catenin and *P* = 0.025 for the interaction test between β-catenin and stage). Further exploratory subgroup analyses of β-catenin membrane staining by standard clinicopathological variables showed that the initial association to survival was also particularly significant for females (OS, Females, *P* = 5.2 × 10^−4^, *n* = 380; Males, *P* = 0.18, *n* = 342; TTR, Females, *P* = 0.047, *n* = 292; Males, *P* = 0.78, *n* = 276). However, interaction tests were not significant.

**Table 6:** **Univariate and multivariate modeling of subgroup analyses by Cox regression (Wald test)**.

Variable	Univariate						Multivariate^a^				
	*P*^a^	HR	95%	*P*	*n*	Events	HR	95% CI	*P*	*n*	Events
**β-CATENIN MEMBRANE STAINING^b^**											
Weak		1					1				
Moderate		8.6	2.86–25.8				3.2	0.97–10.5			
Strong	3.7 × 10^−7^	2	0.73–5.41	5.7 × 10^−8^	134	125	0.92	0.32–2.67	6.7 × 10^−5^	120	112
**E-CADHERIN CYTOSOLIC STAINING^c^**											
Weak		1					1				
Moderate		0.63	0.39–1.03				0.55	0.32–0.93			
Strong	0.033	0.51	0.21–1.19	0.091	273	74	0.36	0.14–0.94	0.024	239	64

*^a^Logrank test for trend*.

*^b^Subgroup analysis for tumor stage IV using overall survival as endpoint, adjusted for age, gender, grade, location, resection, and microsatellite status*.

*^c^Subgroup analysis for tumor stage II using time to recurrence as endpoint, adjusted for age, gender, grade, location, and microsatellite status*.

Decreased cytosolic β-catenin staining was significantly associated with a worse prognosis in both OS (Table 4) and TTR univariate analyses (Figure 3; Table 5).

Multivariate analysis (OS) demonstrated that cytosolic β-catenin expression was an independent prognostic marker (Table 4). Multivariate TTR analysis exhibited a similar trend, but was not statistically significant at the conventional 5% level (Table 5).

Exploratory subgroup analyses (OS) showed a particular age-related effect for younger patients (<69 years of age, *P* = 0.0010, *n* = 246; 69–77 years of age, *P* = 0.62, *n* = 227, 78–96 years of age, *P* = 0.26, *n* = 249). This finding was supported by significant interaction tests (*P* = 0.0025 for β-catenin and *P* = 0.038 for the interaction test). The strata had unfortunately too few patients and events to perform an adequate TTR analysis.

Nuclear β-catenin staining was not associated with prognosis alone or stratified according to standard clinicopathological variables (Figure 3; Tables 4 and 5).

### Loss of E-cadherin independently predicts poor outcome

**Figure 4:**
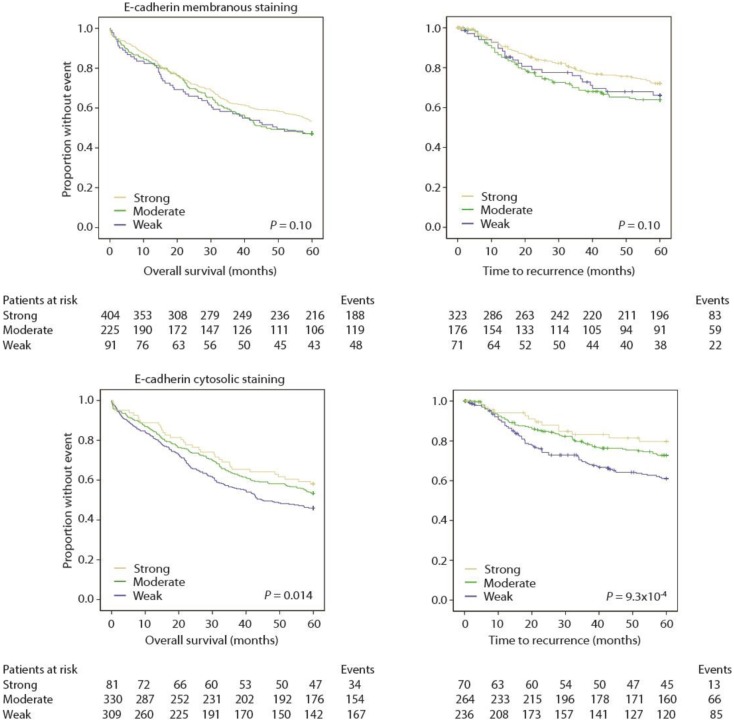
**Weak staining of E-cadherin predicts poor outcome**.

In univariate analyses, there was no significant association between membranous E-cadherin staining and prognosis, but the KM-plots and logrank tests suggest that loss of membranous E-cadherin is associated with a worse prognosis (Figure 4; Tables 4 and 5).

However, there was a significant association between loss of cytosolic E-cadherin and a worse prognosis, both employing OS (Table 4) and TTR (Table 5) as an endpoint (Figure 4).

When standard clinicopathological variables were adjusted for by Cox modeling, cytosolic E-cadherin staining was still an independent prognostic biomarker in TTR analysis (Table 5), but not in OS analysis (Table 4). Stratification by stage suggested that the effect was limited to stage II [*P* = 0.046, *n* = 299 with 111 events (OS) and *P* = 0.033, *n* = 279 with 74 events (TTR) (Table 6)]. However, interaction tests were not significant. Further exploratory subgroup analyses did not pinpoint other effects.

### SOX9 is not associated with prognosis

**Figure 5:**
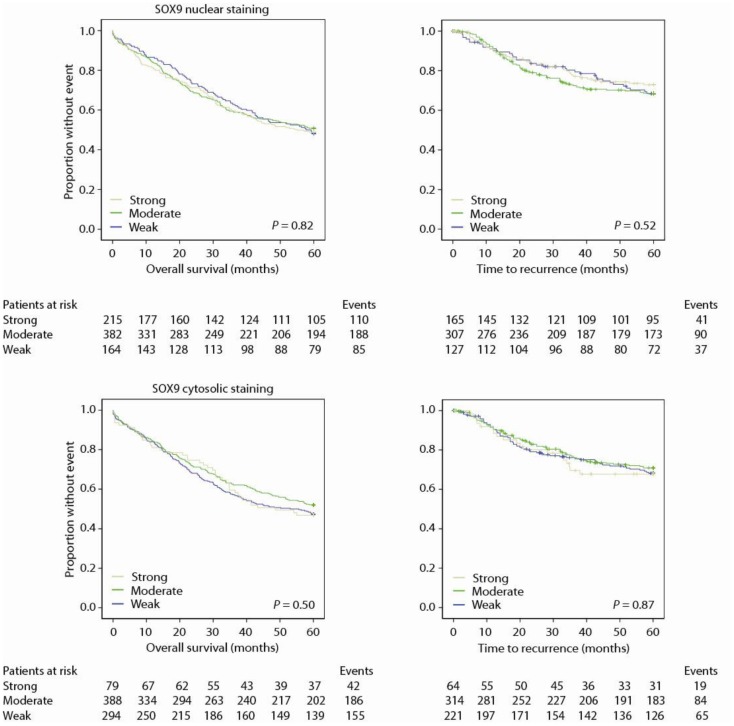
**SOX9 does not predict disease outcome**.

Neither nuclear nor cytosolic staining of SOX9 protein was associated with prognosis, neither unstratified nor stratified on subgroups (Figure 5; Tables 4 and 5).

## Discussion

In the present study, we took advantage of the high-throughput capabilities of the TMA technology to evaluate the prognostic value of β-catenin, E-cadherin, and SOX9 protein expression in a large consecutive population-representative series of primary CRCs. We found that loss of β-catenin or E-cadherin protein expression in tumors was associated with worse disease outcome. This result fits well with a large body of evidence which demonstrates that β-catenin and E-cadherin are often down-regulated in cancer (17, 18, 51–54), reflecting the invasive properties of cancer cells and follows logically from a malignant cancer cell’s need to detach from neighboring cells through decoupling of adherens junctions and activation of Wnt/β-catenin signaling in order to invade neighboring tissue (55). SOX9, on the other hand, was shown to not carry any prognostic information.

In normal colonic epithelia, both β-catenin and E-cadherin are predominantly expressed at the cell membrane (22, 25, 26, 56, 57), and many previous studies have analyzed the prognostic value of their altered expression in tumors, but the reported results are highly divergent (18, 19). While some studies have reported worse outcome for patients with low expression of β-catenin in the nucleus and other compartments (27–30), others have reported no difference in prognosis (26, 31, 43), and yet others have found that strong expression is associated with poor outcome (22, 32–34). The literature on E-cadherin is also marked by conflicting results, where some groups have reported no prognostic effect of altered E-cadherin expression (35, 36), whereas others have reported that patients with low E-cadherin expression have a poor prognosis (37, 38, 58).

Likely reasons for these discrepant results may be that the large majority of the published studies were carried out retrospectively in small series with different patient inclusion criteria, and the analytical approaches employed vary greatly, particularly in reference to clinical endpoints, primary antibodies, immunohistochemical scoring systems, cutoff thresholds, and reporting of statistical methodology. The selection, quality, and representativeness of a patient series may also bias the results.

The patients in the present study were consecutively enrolled from a geographically defined catchment area, and all relevant clinical data were prospectively registered. Repeated quality controls have been performed for the hospital records to ensure high quality of these data. Furthermore, completeness of the series was verified against the Cancer Registry of Norway where all cancer diagnoses in Norway are recorded. Hence, the series can be considered to be population-representative and of a size that allows for stratification and subgroup analyses.

Recently, a meta-analysis assessed the prognostic significance of β-catenin protein expression in CRC and concluded that nuclear expression was significantly associated with a poor prognosis, while cytoplasmic expression was not associated to prognosis (59). However, when we repeated the meta-analysis using their input data, we found a significant publication bias, and when we adjusted for this using a trim-and-fill approach (60), we found that there was no prognostic effect of nuclear β-catenin expression (Figure S2 in Supplementary Material), which is in agreement with our finding. Furthermore, two other large studies that did not find any effect of nuclear β-catenin were not included in the meta-analysis (22, 30). An overview of all the main findings in studies having sample sizes above 200 is summarized in Table S1 in Supplementary Material.

Even though the corrected meta-analysis suggests that there is no prognostic value in assessing nuclear β-catenin expression, one cannot exclude an undefined functional and prognostic relationship among membranous, cytosolic, and nuclear β-catenin protein expression. Furthermore, the phosphorylation status of β-catenin has also been shown to carry prognostic information (26). More quantitative tools are needed to determine these relationships exactly. It may also be functionally relevant to investigate the expression of β-catenin at the tumor invasion front where it has been shown to play an important role in the process of EMT (55, 61–63).

Potential benefit from combining the markers β-catenin, E-cadherin, and SOX9 was explored, but this did not improve stratification of patients, likely due to the high correlation between β-catenin and E-cadherin and the lack of prognostic information carried by SOX9.

Notably, we found that MSI tumors exhibit a significantly lower expression of β-catenin, E-cadherin, and SOX9 protein than MSS tumors. Similarly, right-sided tumors showed lower protein expression compared to left-sided and rectal tumors, although this is likely dependent on the higher level of MSI tumors on the right side. Similar correlations have been reported by other groups (22, 30, 31, 64, 65). The low expression of β-catenin, E-cadherin, and SOX9 in MSI tumors suggests that the Wnt/β-catenin pathway may be less important in these tumors. On the other hand, both MSI and MSS tumors are dependent on active Wnt/β-catenin signaling. Recent studies have suggested that tumorigenic Wnt/β-catenin signaling may be subject to dose- and tissue-dependent regulation through the existence of different *APC* genotypes in right- and left-sided tumors (66). *APC*-mutations reflecting differential inactivation have been documented in independent tumor series (67–69), supporting the “just-right”-hypothesis that different thresholds exist for optimal tumorigenic Wnt/β-catenin signaling (66, 70), which may explain the observed differences in staining between MSI and MSS tumors. Moreover, it has been reported that small absolute changes (but rather relevant fold-changes) in β-catenin levels may have significant effect on Wnt/β-catenin signaling (71). Hence, a lower expression of these proteins may not necessarily indicate that the functional effects are different from in tumors with a higher absolute protein expression.

There was also a positive correlation between the three biomarkers and the histopathologic grade of the tumor. This is in accordance with the assumption that tumors with lower differentiation grade have a more mesenchymal phenotype with down-regulated levels of adherens junctions and presumably lower activity of particular components of the Wnt/β-catenin signaling pathway. We also note that the nuclear staining of SOX9 does not correlate significantly to MSI and histopathologic grade, which may suggest different biological roles for SOX9 in these two compartments.

In our dataset, SOX9 did not carry any prognostic information contrasting with the finding by Lü et al., which reported that strong SOX9 protein expression was an independent adverse prognostic biomarker in a Chinese patient population of 188 primary CRCs with complete resection (43). Selection bias and preanalytical variability may partly explain the lack of accordance as their samples were retrieved from three different hospitals and also constitute a considerably smaller series in total. Significant population effects may also exist due to genetic differences between populations.

Limitations to our study are primarily related to the previously mentioned shortcomings of protein analyses by immunohistochemistry being subject to preanalytical variability, tumor heterogeneity, and subjective scoring systems limiting its reproducibility. The two former are to a certain degree compensated for by using a large sample series, while the latter can only be assessed properly by independent validations. We are also aware that stage migration, due to more patients being classified to higher stages while diagnostic methods have become more sensitive, may bias our assessments of stage specific survival (45).

TNM-stage classification is well-established to assess CRC prognosis. Survival varies considerably between stages, at about 90% for localized disease compared to about 10% for metastatic disease (72), although prognosis may vary significantly within stages. Adjuvant chemotherapy is offered as standard treatment to high-risk stage II patients and stage III patients below 75 years of age. However, up to one-third of stage II patients relapse (3) and elderly patients seem to benefit from adjuvant therapy (4, 5), justifying a need for prognostic biomarkers that can identify high- and low-risk patients in these subgroups. Recently, MSI was introduced into clinical guidelines as a marker for improved prognosis that also likely predicts lack of response to 5-FU monotherapy (73) (www.nccn.org). In our study, strong versus weak β-catenin membrane expression showed the clearest stratification of patients into poor and good prognostic groups (Table 4). Unfortunately, subgroup analysis by stage demonstrated that this effect was evident neither in stage II nor in stage III, but rather in stage IV, suggesting that stage IV patients with low β-catenin membrane expression may benefit from a more *or* less intensive treatment depending on their health condition. Cytosolic E-cadherin on the other hand, might have some prognostic value for stratification of stage II patients. Independent validations are warranted to confirm these results. If validated, we believe β-catenin and E-cadherin may serve as valuable biomarkers in a panel of biomarkers. They are not likely to separate high- and low-risk patient groups with sufficient precision for a clinical test as sole biomarkers.

In summary, nuclear β-catenin protein expression lacks prognostic value for CRC, while decreased expression of both membranous and cytosolic E-cadherin and β-catenin are associated with worse outcome among primary CRC patients, having potential to serve as biomarkers in stage II and IV CRC, respectively.

## Author Contributions

Jarle Bruun participated in the study design, performed IHC experiments and scoring, interpreted all results, performed all statistics, and drafted the manuscript. Matthias Kolberg participated in IHC experiments and in the statistical analyses and did an independent scoring of results. Arild Nesbakken collected the patient samples and provided the clinical data. Jahn M. Nesland performed quality control of the IHC analysis and carried out independent scoring of a subset of the data. Aud Svindland performed morphological identification of qualified tumor areas for TMA construction. Ragnhild A. Lothe conceived and coordinated the study, was responsible for the study design, and participated in discussion of results and in the drafting of the manuscript. All authors participated in manuscript writing and in scientific discussions.

## Conflict of Interest Statement

The authors declare that the research was conducted in the absence of any commercial or financial relationships that could be construed as a potential conflict of interest.

## Supplementary Material

The Supplementary Material for this article can be found online at http://www.frontiersin.org/Journal/10.3389/fonc.2014.00118/abstract
