# Supplementary material for: Prognostic Significance of β-Catenin, E-Cadherin, and SOX9 in Colorectal Cancer: Results from a Large Population-Representative Series
Source: Front Oncol. 2014 May 21;4:118. doi: 10.3389/fonc.2014.00118 (PMC4033250; doi:10.3389/fonc.2014.00118)
Supplement: Supplementary file 1 [file Data_Sheet1.PDF]

Supporting information for article: "Prognostic significance of β-catenin, E-cadherin and SOX9 in colorectal cancer: results from a large population-representative series"

Jarle Bruun[1,2], Matthias Kolberg[1,2], Jahn M. Nesland[5], Aud Svindland[4,5], Arild Nesbakken[2,4,6] and Ragnhild A. Lothe[1,2,3]*

	Item to be reported	Page no.
**INTRODUCTION**		
1	State the marker examined, the study objectives, and any pre-specified hypotheses.	4
**MATERIALS AND METHODS**		
Patients		
2	Describe the characteristics (e.g., disease stage or co-morbidities) of the study patients, including their source and inclusion and exclusion criteria.	5-8
3	Describe treatments received and how chosen (e.g., randomized or rule-based).	5-8
Specimen characteristics		
4	Describe type of biological material used (including control samples) and methods of preservation and storage.	5
Assay methods		
5	Specify the assay method used and provide (or reference) a detailed protocol, including specific reagents or kits used, quality control procedures, reproducibility assessments, quantitation methods, and scoring and reporting protocols. Specify whether and how assays were performed blinded to the study endpoint.	5-7
Study design		
6	State the method of case selection, including whether prospective or retrospective and whether stratification or matching (e.g., by stage of disease or age) was used. Specify the time period from which cases were taken, the end of the follow-up period, and the median follow-up time.	5
7	Precisely define all clinical endpoints examined.	7
8	List all candidate variables initially examined or considered for inclusion in models.	7
9	Give rationale for sample size; if the study was designed to detect a specified effect size, give the target power and effect size.	5
Statistical analysis methods		
10	Specify all statistical methods, including details of any variable selection procedures and other model-building issues, how model assumptions were verified, and how missing data were handled.	7-8
11	Clarify how marker values were handled in the analyses; if relevant, describe methods used for cutpoint determination.	6-7
**RESULTS**		
Data		
12	Describe the flow of patients through the study, including the number of patients included in each stage of the analysis (a diagram may be helpful) and reasons for dropout. Specifically, both overall and for each subgroup extensively examined report the numbers of patients and the number of events.	6, 12-19
13	Report distributions of basic demographic characteristics (at least age and sex), standard (disease-specific) prognostic variables, and tumor marker, including numbers of missing values.	8
Analysis and presentation		
14	Show the relation of the marker to standard prognostic variables.	14-17
15	Present univariable analyses showing the relation between the marker and outcome, with the estimated effect (e.g., hazard ratio and survival probability). Preferably provide similar analyses for all other variables being analyzed. For the effect of a tumor marker on a time-to-event outcome, a Kaplan-Meier plot is recommended.	13-19
16	For key multivariable analyses, report estimated effects (e.g., hazard ratio) with confidence intervals for the marker and, at least for the final model, all other variables in the model.	14-17
17	Among reported results, provide estimated effects with confidence intervals from an analysis in which the marker and standard prognostic variables are included, regardless of their statistical significance.	14-17
18	If done, report results of further investigations, such as checking assumptions, sensitivity analyses, and internal validation.	7
**DISCUSSION**		
19	Interpret the results in the context of the pre-specified hypotheses and other relevant studies; include a discussion of limitations of the study.	20-22
20	Discuss implications for future research and clinical value.	22

Supplementary Table 1. The REMARK checklist.

Number of CRC	Comment	Prognostic effect of nuclear β-catenin	Prognostic effect of cytoplasmic β-catenin	Prognostic effect of membrane β-catenin	Ref		PMID
386	stage II & III	no effect	nd	nd	Belt EJ,	Ann Surg Oncol. 2012	22311118
355		Increased levels in primary tumors with synchronous liver metastases	nd	nd	Cheng H	Diagn Pathol. 2011	22053859
955	stage I-IV	high in males, high in rectum, high in MSS, high in braf neg negative. Good prognosis in obese	high in rectum, high in MSS, high in braf negative	high in rectum, high in MSS, high in braf negative	Morikawa T,	JAMA. 2011	21521850
486		Correlated to T, stage, pN, pM liver,age,	nd	nd	Wang L,	Ann Surg Oncol. 2011	21207157
470		no effect on cdk8	correlates with CDK8	correlates with CDK8	Firestein R,	Int J Cancer. 2010	19790197
264		Associated with MMP9 in invasive front			Delektorskaya VV,	Bull Exp Biol Med. 2008	19526105
1420		Minor effect on survival in MSS	no effect	Loss associated with poor survival in MSS	Lugli A<br>Zlobec, I	Histopathology. 2007<br>Virchows Arch. 2007	17448021<br>17674041
269	Rectal		low levels associated with metastatic disease	low levels associated with metastatic disease	Fernebro E	Int J Cancer. 2004	15300804
205		Accumulation at invasive front gives poor ooutcome			Baldus SE,	Clin Cancer Res. 2004	15102686
202		Accumulation at invasive front in advanced stages			Ougolkov AV,	Gastroenterology. 2002	11781281
650	Phospho β-catenin	Good prognosis			Chung GG,	Clin Cancer Res. 2001	11751495

832	high in left/rectum, low in msi/brafmut/cimp-h	high in left/rectum, low in msi/brafmut/cimp-h	high in left/rectum, low in msi/brafmut/cimp-h	Kawasaki T,<br>Baba, Y<br>Baba, Y	Neoplasia 2007<br>Cancer Epidemiol Biomarkers Prev. 2010<br>Clin Cancer Res. 2009	17710160<br>20200425<br>19584150
557	neg nucl/cyt ->poor survival (esp for stage 3-4)	neg nucl/cyt ->poor survival (esp for stage 3-4)		Wangefjord S,	Diagn Pathol. 2013	23337059

Supplementary Table 2. Overview of literature on β-catenin (n > 200).

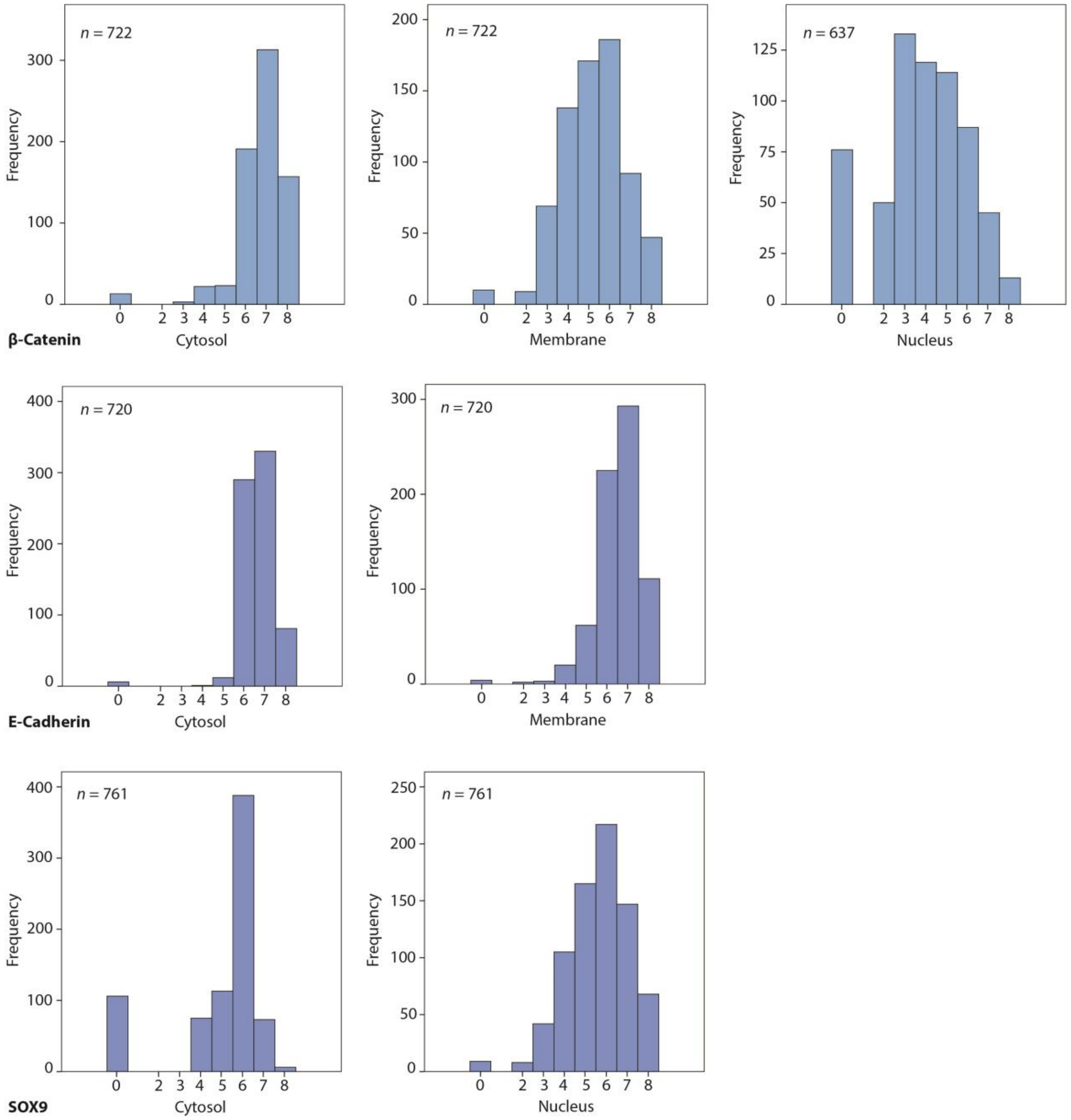

Supplementary Figure 1. Distributions of tumor staining scores for β-catenin, E-cadherin and SOX9 protein expression. Almost all tumors exhibited some degree of membranous (99% 712/722) and cytosolic β-catenin staining (98% 709/722), while nuclear staining was evident in 88% (561/637) of the tumors. Similar membranous and cytosolic expression patterns were observed for E-cadherin (99%, 716/720 and 99%, 714/720, respectively). Membranous staining was generally more heterogeneous than cytosolic staining, for both β-catenin and E-cadherin. For SOX9, 86% (655/761) of tumors were positive for cytosolic staining and 99% (752/761) were positive for nuclear staining.

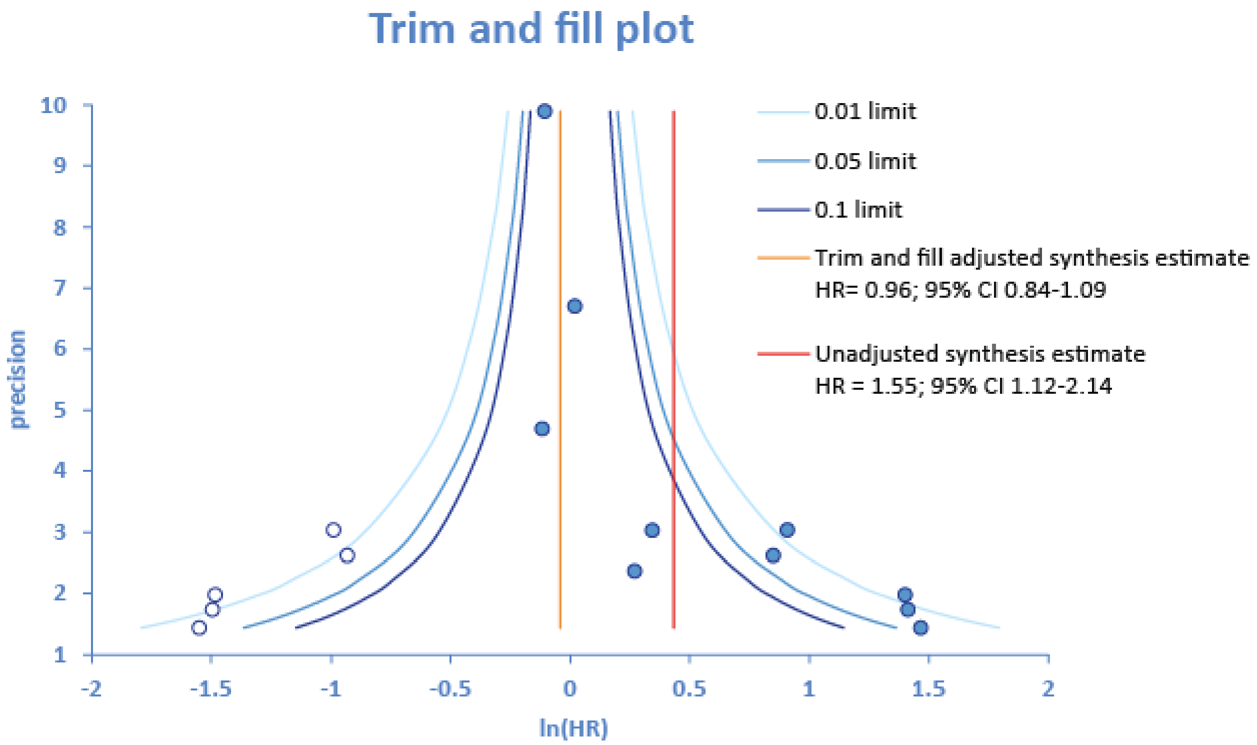

Supplementary Figure 2. Reassessment of a recent meta-analysis (including n=10 studies, blue circles) which concluded that nuclear β-catenin expression was significantly associated with a poor prognosis (red line, HR 1.55, 95% CI 1.12-2.14). When we adjusted for publication bias using a trim-and-fill approach (introducing five complementary small studies (open circles)), we found that there was no prognostic effect of nuclear β-catenin expression (orange line, HR 0.96, 95% CI 0.84-1.09).
